# Losartan-associated toothache in a patient with diabetes and hypertension: a hypothesis-generating case report

**DOI:** 10.3389/fphar.2025.1679182

**Published:** 2025-10-01

**Authors:** Li Yuan, Mingxue Tang, Shuaiju Liao, Dan Xiang, Tao Liu, Yangtian Wang

**Affiliations:** ^1^ Department of endocrinology, Nanjing University Medical School Affiliated Taikang Xianlin Drum Tower Hospital, Nanjing, China; ^2^ School of Chinese Medicine, Nanjing University of Chinese Medicine, Nanjing, China; ^3^ Department of Health and Civil Affairs, Administrative Committee of Nanjing Jiangbei New Area, Nanjing, China

**Keywords:** losartan, toothache, adverse drug reaction, pharmacovigilance, orofacial pain

## Abstract

**Background:**

Angiotensin II receptor blockers (ARBs) are standard of care for hypertensive patients, particularly those with type 2 diabetes mellitus (T2D). Although generally well tolerated, rare adverse drug reactions can be overlooked. To our knowledge, no prior case report up to 2025 has described isolated toothache attributable to losartan.

**Case Presentation:**

A 45-year-old man with T2D and essential hypertension developed a reproducible, diffuse maxillary toothache ≈30 min after each 50 mg dose of losartan potassium. Oral examination and panoramic radiography found no odontogenic pathology; routine laboratory tests were unremarkable. Symptoms resolved upon drug withdrawal and reappeared with supervised re-challenge; no recurrence occurred after substitution with candesartan. The Naranjo algorithm yielded a score of 7, supportive of a drug-related event, though causality cannot be proven in a single case.

**Conclusion:**

This observation is hypothesis-generating and suggests that losartan may rarely be associated with toothache. Recognizing this possibility may prevent unnecessary dental procedures and guide ARB substitution when appropriate. Systematic pharmacovigilance and mechanistic research are warranted.

## 1 Introduction

Twin epidemics, one prescription pad. Hypertension and type 2 diabetes (T2D) travel in lockstep: up to 50%–80% of people with T2D carry a simultaneous hypertension diagnosis, and the proportion is still climbing globally (Makarem et al., 2025). Between 1999 and 2018, the prevalence of this double-hit in U.S. adults alone doubled from 6% to 12% (Goldberg et al., 1995). Their coexistence not only accelerates cardiovascular and renal complications but also forces clinicians to reach early for renin–angiotensin system (RAS) blockers to achieve the synergistic goals of blood-pressure control, renoprotection and metabolic safety.

The RAS exerts its biological effects mainly via angiotensin II. AT_1_ receptor activation drives vasoconstriction, sodium retention, and pro-inflammatory remodeling, whereas AT_2_ receptor activation mediates vasodilation and counter-regulatory effects. ARBs such as losartan selectively block AT_1_ receptors, thereby lowering blood pressure and protecting renal function (DailyMed, 2025; Jain and Deshpande, 2023).

Losartan—the prototype angiotensin II type 1 receptor blocker (ARB)—has been prescribed for 3 decades because it works, is kidney-friendly and generally well tolerated. Large registries and reference texts assign it an adverse-event profile indistinguishable from placebo save for well-known issues such as dizziness, hyperkalaemia and rare angio-oedema (Schlienger et al., 1996; Balakumar et al., 2015). Dig a little deeper, however, and the official pharmacovigilance sheets quietly list “dental pain” under “frequency not reported” (Drugs, 2025). In everyday practice, that single line is easy to scroll past.

The literature does little to flesh out the risk signal. PubMed surfaces just a handful of ARB-related oral problems—aphthous ulcers, glossitis, reversible ageusia, even angio-oedema of the tongue—but no stand-alone report of toothache attributable solely to losartan (Balogh et al., 2021; Souza et al., 2007). Whether the symptom is truly rare, under-recognised or simply mislabelled as “idiopathic facial pain” remains unknown.

Here we throw the spotlight on a gap in the safety record. We present what appears to be the first documented case of isolated, reproducible tooth pain triggered by therapeutic-dose losartan in a patient living with both diabetes and hypertension. By walking through the chronology, differential work-up and Naranjo causality scoring, we aim to.

1. Expand the catalogue of ARB-associated oral adverse reactions;
2. Offer practical guidance for clinicians faced with unexplained dental pain in multimorbid patients; and
3. Stimulate mechanistic research into how AT_1_-receptor blockade might influence trigeminal nociception or pulpal microcirculation.

## 2 Case Presentation

A 45-year-old male patient with an 8-year history of type 2 diabetes and a 5-year history of essential hypertension was switched from amlodipine 5 mg qd to losartan potassium 50 mg qd for better reno-protective control. Within ≈30 min of the first dose the patient experienced a diffuse, throbbing maxillary toothache rated 7/10 on the VAS; the pain abated completely ≈2 h after omitting the next dose. Over the following week the toothache recurred at the same post-dose interval every day, with no visible dental lesions on panoramic radiography or intra-oral examination. This case is reported in accordance with the CARE guidelines (Gagnier et al., 2014).

Self-discontinuation of losartan led to immediate and sustained relief, while a monitored re-challenge reproduced the pain (VAS 8/10) within 25 min. The patient had been receiving metformin (1 g bid) and atorvastatin (20 mg qhs) chronically. No analgesics, antibiotics or other concomitant medications were used during the observation period. Laboratory tests—including serum potassium, CRP, fasting glucose, HbA1c, and eGFR—were unremarkable.

A dentist evaluated the patient in conjunction with the medical team and found no signs of pulpitis, abscess, periodontitis, or trigeminal neuralgia. Pulp vitality testing was not performed, which we acknowledge as a limitation. Substitution with candesartan 8 mg qd controlled blood pressure (132/80 mmHg) without recurrence of dental symptoms during a 3-month follow-up (Table 1).

**TABLE 1:** Clinical summary table–losartan-associated toothache.

Item	Details
Patient characteristics	45-year-old male; T2D (8 years); hypertension (5 years)
Concomitant medications	Metformin 1 g bid; atorvastatin 20 mg qhs; no analgesics/antibiotics
Suspected drug	Losartan potassium 50 mg qd
Symptom onset	Diffuse maxillary toothache (VAS 7/10) ≈30 min post-dose
Diagnostic tests	Oral exam and panoramic radiography: no pathology; labs: unremarkable
Dental evaluation	Specialist review: no pulpitis, abscess, or periodontal disease
De-challenge	Complete resolution
Re-challenge	Recurrence (VAS 8/10) within 25 min
Alternative antihypertensive	Candesartan 8 mg qd; no recurrence (3 months)
Causality assessment	Naranjo score: 7 (probable, supportive not definitive)

## 3 Discussion

### 3.1 How uncommon is losartan-linked toothache?

Losartan has been marketed since 1995 and remains front-line therapy for diabetic hypertension. Yet in its publicly available label the term “dental pain” is buried under “frequency not reported,” meaning fewer than one spontaneous report per 10,000 treated patients (Drugs, 2025). A targeted PubMed sweep retrieves only a handful of oral ADR case reports—aphthous ulcer (Jain and Deshpande, 2023), glossitis, reversible ageusia (Schlienger et al., 1996), and angio-oedema (Balakumar et al., 2015)—but no prior publication that singles out isolated toothache. Against this backdrop, the reproducible, de-challenge/re-challenge–positive episode we describe likely represents a rare but plausible pharmacovigilance signal rather than diagnostic oversight. Importantly, as a single case report, this observation should be considered hypothesis-generating and not confirmatory.

### 3.2 Plausible pathophysiological pathways

1. Pulpal microcirculation. Dental pulp expresses a local renin–angiotensin system (RAS); AT_1_ and AT_2_ receptor mRNA levels rise during experimentally induced pulpitis in rats (Souza et al., 2007). Ang II modulates proliferation and cytokine release in stem cells of the apical papilla (Pizzatto et al., 2020), reinforcing the concept that RAS blockers could disturb pulpal homeostasis. Sudden vasodilation after AT_1_ blockade might provoke transient ischemia–reperfusion of the pulp, experienced clinically as throbbing pain.
2. Sensory-nerve modulation. AT_1_ and AT_2_ receptors are distributed throughout pain-relay nuclei and peripheral sensory ganglia, including the trigeminal system (Balogh et al., 2021). Experimental work demonstrated that Ang II enhances acid-sensing ion-channel currents in dorsal-root and trigeminal neurons via AT_1_R activation, thereby amplifying nociceptive firing (Xu et al., 2025). Abrupt blockade of this tonic Ang II signal by losartan could paradoxically lower the activation threshold of adjacent pain circuits, unmasking toothache without overt inflammation.
3. Idiosyncratic immune/hypersensitivity response. Other ARBs have elicited aphthous ulcers and oral angio-oedema, suggesting a mast cell–mediated or neuro-immune mechanism (Jain and Deshpande, 2023; Balakumar et al., 2015). Although our patient showed no mucosal swelling, the rapid onset and offset of pain remain compatible with a hypersensitivity-like phenotype, which cannot be entirely excluded.

### 3.3 Clinical considerations

• Suspect the drug, not the pulp. A pain pattern that occurs consistently after each dose and vanishes on withdrawal should trigger a careful medication review before invasive dental procedures are considered.
• Use structured causality assessment. The Naranjo triad (Table 2)—timing, de-challenge, and re-challenge—helped support the association in our case. However, the tool is supportive rather than definitive in single-patient scenarios (Naranjo et al., 1981).
• Switch rather than stop consistent with the 2024 ESC Hypertension Guidelines (McEvoy et al., 2024). Guideline-concordant management allows lateral substitution within the ARB family. In our patient, candesartan maintained blood-pressure control without relapse of toothache.

**TABLE 2:** Naranjo causality assessment.

#	Question	Response	Score
1	Previous conclusive reports?	No	0
2	Did the event appear after drug given?	Yes	+2
3	Did it improve on de-challenge?	Yes	+1
4	Did it reappear on re-challenge?	Yes	+2
5	Alternative causes ruled out?	Yes	+2
6	Did it reappear with placebo?	Not done	0
7	Toxic drug levels documented?	Not applicable	0
8	Dose–response observed?	Not assessed	0
9	Similar reaction before?	No	0
10	Objective evidence (lab)?	No (clinical only)	0
Total			7 (Probable)

### 3.4 Pharmacovigilance context

A review of FAERS (US Food and Drug Administration, 2025) and EudraVigilance (European Medicines Agency, 2025) revealed no clearly documented isolated cases of losartan-induced toothache. This absence emphasizes both the rarity of the observation and the need for systematic reporting to strengthen pharmacovigilance databases (Table 3).

**TABLE 3:** Comparative overview of losartan adverse effects.

Adverse effect	Frequency	Notes
Dizziness	Common	Often dose-related
Hyperkalaemia	Common	Especially in CKD/diabetes
Hypotension	Common	Usually postural
Angioedema	Rare	Documented with ARBs/ACEIs
Aphthous ulcers	Very rare	Isolated reports (Jain and Deshpande, 2023)
Glossitis	Very rare	Occasional
Reversible ageusia/dysgeusia	Very rare	Lancet 1996 (Schlienger et al., 1996)
Toothache (this case)	Not previously reported up to 2025	Hypothesis-generating ADR

### 3.5 Limitations and research agenda

This report is limited by its single-patient design, absence of pulp vitality testing, and lack of dose–response data. Future research should explore:

• Pulpal laser-Doppler flowmetry during ARB exposure,
• Electrophysiologic mapping of trigeminal AT_1_/ASIC interactions, and
• Disproportionality analyses in large pharmacovigilance datasets for “tooth pain + losartan.”

## 4 Conclusion

In summary, this case provides evidence that losartan, despite its established safety, may occasionally be associated with reproducible toothache that resolves on withdrawal and recurs on re-challenge. While the Naranjo score of 7 suggests a probable ADR, the single-case nature limits confirmatory conclusions. Thus, this report should be interpreted as raising a pharmacovigilance signal rather than proving causality.

Clinicians should maintain vigilance when encountering unexplained dental pain, consider medication history before invasive interventions, and recognize that lateral ARB substitution is often feasible. Systematic reporting and further research into RAS modulation of orofacial pain will refine our understanding and may enhance patient safety.

## Data Availability

The original contributions presented in the study are included in the article/supplementary material, further inquiries can be directed to the corresponding authors.
